# Supplementary material for: Protective role of neuronal and lymphoid cannabinoid CB2 receptors in neuropathic pain
Source: eLife. 2020 Jul 20;9:e55582. doi: 10.7554/eLife.55582 (PMC7384863; doi:10.7554/eLife.55582)
Supplement: Transparent reporting form [file elife-55582-transrepform.pdf]

eLIFE
Westbrook Centre, Milton Road
Cambridge CB4 1YG
UK
P 01223 855340
W elifesciences.org
T @elife

# *eLife's* transparent reporting form

We encourage authors to provide detailed information *within their submission* to facilitate the interpretation and replication of experiments. Authors can upload supporting documentation to indicate the use of appropriate reporting guidelines for health-related research (see EQUATOR Network), life science research (see the BioSharing Information Resource), or the ARRIVE guidelines for reporting work involving animal research. Where applicable, authors should refer to any relevant reporting standards documents in this form.

If you have any questions, please consult our Journal Policies and/or contact us: editorial@elifesciences.org.

**Sample-size estimation**

- You should state whether an appropriate sample size was computed when the study was being designed
- You should state the statistical method of sample size computation and any required assumptions
- If no explicit power analysis was used, you should describe how you decided what sample (replicate) size (number) to use

Please outline where this information can be found within the submission (e.g., sections or figure legends), or explain why this information doesn't apply to your submission:

Sample size was based on previous studies in our laboratory using comparable behavioral approaches (Bura et al., Eur J Pain 2013; La Porta et al., Pain 2015; Bura et al., Eur J Pain 2018). This information is included in Materials and Methods section, end of the first paragraph of Animals subsection.

**Replicates**

- You should report how often each experiment was performed
- You should include a definition of biological versus technical replication
- The data obtained should be provided and sufficient information should be provided to indicate the number of independent biological and/or technical replicates
- If you encountered any outliers, you should describe how these were handled
- Criteria for exclusion/inclusion of data should be clearly stated
- High-throughput sequence data should be uploaded before submission, with a private link for reviewers provided (these are available from both GEO and ArrayExpress)

Please outline where this information can be found within the submission (e.g., sections or figure legends), or explain why this information doesn't apply to your submission:

eLife Sciences Publications, Ltd is a limited liability non-profit non-stock corporation incorporated in the State of Delaware, USA, with company number 5030732, and is registered in the UK with company number FC030576 and branch number BR015634 at the address Westbrook Centre, Milton Road Cambridge CB4 1YG, UK | March 2019

The first experiment assessing the effects of the different doses of JWH133 was performed 3 times (Figure 1). The second experiment assessing the effects of JWH133 in CB2 constitutive knockout mice was performed 4 times (Figure 2). The third experiment assessing the effects of JWH133 in CB2 conditional knockout mice was performed 5 times (Figure 3). The bone-marrow transplantation experiment was conducted once (Figure 4). The experiment assessing the effects of lymphocyte depletion on JWH133 self-administration was performed once (Figure 5). This information is included in Materials and Methods section, end of first paragraph of Animals subsection.

Number of biological replicates (mice) are specified in each figure legend, and technical replicates (values for immunohistochemistry sections and qPCR replicates) are included the Source data file in their respective RawData sheet.

No outliers were excluded for the statistical analysis.

**Statistical reporting**

- Statistical analysis methods should be described and justified
- Raw data should be presented in figures whenever informative to do so (typically when N per group is less than 10)
- For each experiment, you should identify the statistical tests used, exact values of N, definitions of center, methods of multiple test correction, and dispersion and precision measures (e.g., mean, median, SD, SEM, confidence intervals; and, for the major substantive results, a measure of effect size (e.g., Pearson's r, Cohen's d)
- Report exact p-values wherever possible alongside the summary statistics and 95% confidence intervals. These should be reported for all key questions and not only when the p-value is less than 0.05.

Please outline where this information can be found within the submission (e.g., sections or figure legends), or explain why this information doesn't apply to your submission:

Details of the statistical tests used are provided in the Statistics subsection of the Materials and Methods section.
Mean, SEM and significance level are plotted to facilitate interpretation of the data.
Source data file contains raw numerical data for each data point in the figures, mean, SD, SEM, and also the results of Statistical tests used in their respective "Stats" Sheets, confidence intervals, exact values of N, exact p-values and results of Bonferroni correction when appropriate.

(For large datasets, or papers with a very large number of statistical tests, you may upload a single table file with tests, Ns, etc., with reference to sections in the manuscript.)

**Group allocation**

- Indicate how samples were allocated into experimental groups (in the case of clinical studies, please specify allocation to treatment method); if randomization was used, please also state if restricted randomization was applied
- Indicate if masking was used during group allocation, data collection and/or data analysis

Please outline where this information can be found within the submission (e.g., sections or figure legends), or explain why this information doesn't apply to your submission:

Whenever possible, animals were randomly assigned to their experimental condition, and experiments and measurements were performed blinded for pharmacological and surgical conditions, genotype or antibody treatments, as stated in Materials and methods section, end of Animals subsection.

**Additional data files ("source data")**

- We encourage you to upload relevant additional data files, such as numerical data that are represented as a graph in a figure, or as a summary table
- Where provided, these should be in the most useful format, and they can be uploaded as "Source data" files linked to a main figure or table
- Include model definition files including the full list of parameters used
- Include code used for data analysis (e.g., R, MatLab)
- Avoid stating that data files are "available upon request"

Please indicate the figures or tables for which source data files have been provided:

Source Data files for each figure contain all the raw data and statistical analyses conducted with the IBM SPSS 23 software (Chicago, IL, USA). Data availability statement is included at the end of the manuscript. In addition, the ARRIVE guidelines have been followed and the ARRIVE checklist is provided as supporting documentation.
